# Treatment of Cervicofacial Actinomycosis: A report of 19 cases and review of literature

**DOI:** 10.4317/medoral.19124

**Published:** 2013-05-31

**Authors:** Meshkan Moghimi, Erik Salentijn, Yvette Debets-Ossenkop, K H. Karagozoglu, Tymour Forouzanfar

**Affiliations:** 1 Department of Oral and Maxillofacial Surgery/Oral Pathology, VU University Medical Center/Academic Centre for Dentistry Amsterdam (ACTA), P.O. Box 7057, 1007 MB, Amsterdam, The Netherlands; 2 Department of Medical Microbiology and Infection Control, VU University Medical Center, de Boelelaan 1117, 1081 HV Amsterdam, The Netherlands

## Abstract

Objectives: Actinomycosis is a chronic suppurative granulomatous infection caused by the Actinomyces genus. Orocervicofacial actinomycosis is the most common form of the disease, seen in up to 55% of cases. All forms of actinomycosis are treated with high doses of intravenous penicillin G over two to six weeks, followed by oral penicillin V. Large studies on cervicofacial actinomycosis are lacking. Therefore proper guidelines for treatment and treatment duration are difficult to establish. The aim of this study is to establish effective treatment and treatment duration for orocervicofacial actinomycosis. Study design: A Pubmed and Embase search was performed with the focus on treatment and treatment duration for cervicofacial actinomycosis. The hospital records of all patients presenting to our department with head and neck infection from January 2000 to December 2010 were reviewed, retrospectively. The following data were collected: age, gender, clinical presentation, aetiology, duration of symptoms, microbiological findings, treatment, and duration of treatment. The treatment and treatment duration is subsequently compared to the literature. Results: The literature search provided 12 studies meeting the inclusion criteria. All studies were retrospective in nature. Penicillin or amoxicillin/clavulanic acid are the preferred antibiotic regimens found in the literature. Most of our patients were treated with a combination of penicillin G 12 million units/day and metronidazol 500 mg 3/day, most commonly for a duration of 1 – 4 weeks, being shorter than the 3 – 52 weeks reported in the literature. Conclusion: When actinomycosis is suspected, our review has shown that a surgical approach in combination with intravenous penicillin and metronidazol until clinical improvement is seen, followed by oral antibiotics for 2 – 4 weeks is generally efficient.

** Key words:**Actinomycosis, actinomyces, actinomycosis treatment, cervicofacial infection, actinomycosis diagnosis, head and neck infection.

## Introduction

Actinomycosis is a chronic suppurative granulomatous infection caused by the Actinomyces genus. These are non-spore-forming, anaerobic, or microaerophilic gram-positive (1) bacilli. The pathogenic Actinomyces species are only found in humans, and are commensals of the oropharynx, gastrointestinal tract, and female genital tract (1). The Actinomyces species are generally of low pathogenicity, but can cause disease when there is a portal of entry, typically in the mucosa of the gastrointestinal tract, anywhere from the mouth to the rectum (1). Once these organisms invade tissue, they form tiny clumps, called grains or sulfur granules (2). It is now rare but was common in the preantibiotic era (3).

A. israelli is the most common human pathogen and is found in most clinical presentations (4). Cultures with Actinomyces species are often accompanied by other organisms, such as Actinobacillus actinomycetemcomitans, Eikenella corrodens, Fusobacterium, and Bacteroides species (3). These organisms facilitate infection by establishing a microaerophilic environment (3). Orocervicofacial actinomycosis is the most common form of the disease, seen in up to 55% of cases (5,6). Patients frequently present with chronic soft tissue swelling (4). They can also present with abscesses, woody fibrosis, and sinus discharge of sulfur granules (3). The swellings are firm, and often lead to misdiagnosis of malignancy (4,7). Infection can spread directly to adjacent muscles and bones (8). Especially the mandible is reported to be involved in bone disease (9). According to a study by Brook et al. (10), when diagnosing orocervicofacial actinomycosis, one should also consider the following diagnosis: abscess by other typical bacteria, cyst, neoplasm, tuberculosis, or nocardiosis. Demonstration of Gram-positive filamentous organisms and sulphur granules on histological examination is strongly supportive of a diagnosis of actinomycosis (4). However, granules are not specific to actinomycosis (11). For definitive diagnosis, direct isolation of the organisms from a clinical specimen or from sulphur granules is necessary (4). The most appropriate clinical specimens are samples of pus, tissue, or sulphur granules. Antibiotics are the cornerstone of treatment (4). All forms of actinomycosis are treated with high doses of intravenous penicillin G over two to six weeks, followed by oral penicillin V (10). Surgical treatment may be necessary if there is extensive necrotic tissue, sinus tracts, fistulas, or if patients do not respond to medical treatment (4). It may also be needed if malignancy cannot be excluded (10).

Large studies on cervicofacial actinomycosis are lacking. Therefore proper guidelines for treatment and treatment duration are difficult to establish. The aim of the current study is to map patient demographics, disease characteristics, diagnosis, and treatment in all patients admitted to our department from January 2000 to December 2010 with isolated Actinomyces spp. in their pus. The treatment and treatment duration is subsequently compared to the literature in order to establish a more efficient treatment regimen.

## Material and Methods

The hospital records of all patients presenting with head and neck infection from January 2000 to December 2010 were reviewed, retrospectively. The patients were identified using the hospital database. Only patients with positive microbial culture for Actinomyces spp. were included. The records of 19 patients remained for analysis. The following data were collected: age, gender, clinical presentation, aetiology, duration of symptoms, microbiological findings, treatment, and duration of treatment.

A Pubmed and Embase search was performed. The main focus of this search was to determine treatment and treatment duration for cervicofacial actinomycosis. (Mesh) terms included: ‘actinomycosis’, ‘actinomyces’, ‘cervicofacial’, ‘head’, ‘face’, ‘skull’, ‘neck muscles’. All studies meeting the following criteria were included: only cervicofacial actinomycosis, age > 18 years, case reports describing 2 or more patients with cervicofacial actinomycosis, English language. Cases where actinomycosis was diagnosed in 2 different locations simultaneously were excluded. Only immunocompetent patients were analyzed, excluding patients infected with the human immunodeficiency virus, tuberculosis, non-Hodgkin lymphoma, post-renal transplantation, badly regulated diabetes mellitus, and chronic granulomatous disease. Only studies published after 1990 were included in order to ensure analysis of the most recent treatment modalities, and to subsequently make a comparison to our study population. These criteria left 12 articles for inclusion.

## Results

The study population consisted of 10 males and 9 females with a mean age of 37.2 (SD: ± 17.4) years. The youngest patient was 19 years and the oldest 85 years. There was no significant difference in age between male and female patients. All patients were immunocompetent.

-Symptoms

**Figure 1:**
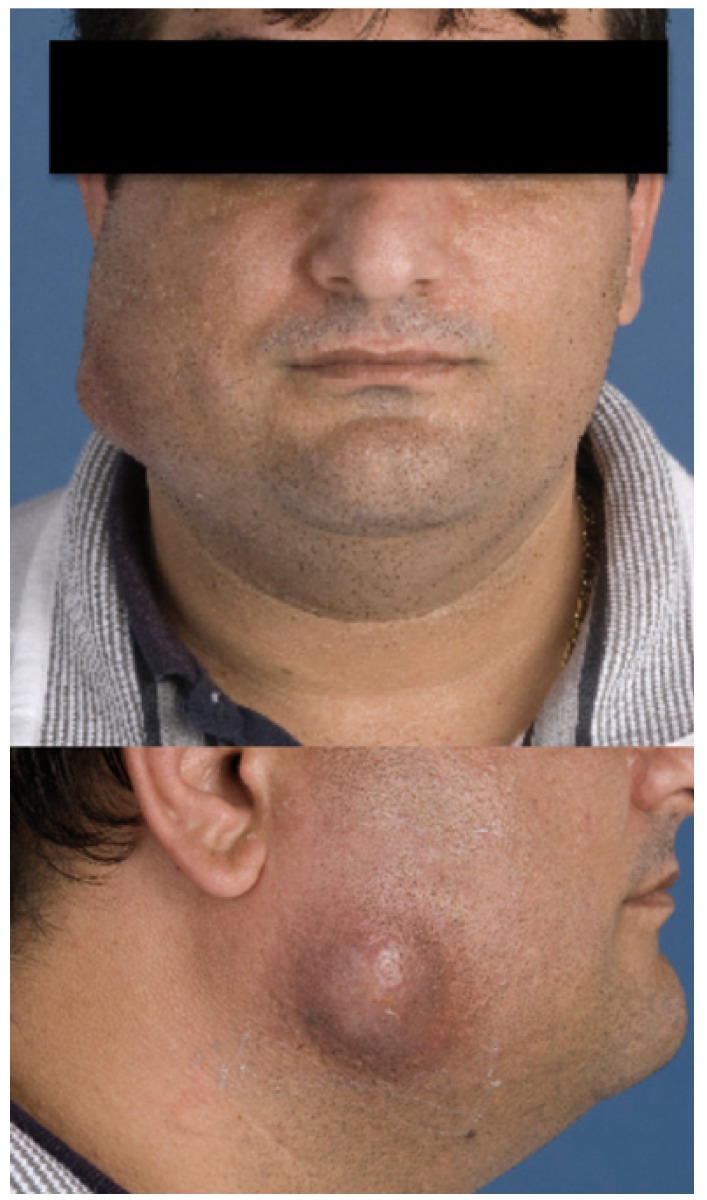
Swelling of the cheek in a patient with actinomycosis. Note the possible beginning of a sinus tract.

All patients presented with a swelling. In 9 (47.4%) cases the swelling was progressive in size and in 3 (15.8%) cases the swelling was recurrent. Seventeen patients (89.5%) complained of pain. Swelling of soft tissue was most frequently located in the mandibular region (6 of 19; 31.6%). Other locations consisted of the cheek (2 of 19; 10.5%) (Fig. 1.), the buccal region (2 of 19; 10.5%), the infratemporal region (1 of 19; 5.3%), the infraorbital region (1 of 19; 5.3%), and the sublingual region (1 of 19; 5.3%). One patient developed necrotizing osteomyelitis of the mandible.

The duration of symptoms prior to admission ranged from 4 days to 1 year (44.2 ± 87.9 days). Most patients (12 of 19; 63.2%) had symptoms for 7 days or less. Three patients had symptoms lasting 1 – 3 months, and 4 patients had symptoms lasting more than 2 months to one year prior to admission. During this period, 9 patients (47.4%) received oral antibiotics, mostly consisting of amoxicillin or amoxicillin/clavulanic acid.

-Aetiology 

The most commonly reported aetiology prior to start of symptoms were removal of a tooth (10 of 19; 52.6%), infected teeth (7 of 19; 36.8%), or trauma (2 of 19; 10.5%).

-Microbiology

The pus of eighteen patients was cultured for the presence of Actinomyces. In one patient, Actinomyces could not be isolated. The pus of this patient was histopathologicaly analyzed and was found positive for Actinomyces sp. The most frequently found species of Actinomyces were A. meyeri (4 of 17; 23.5%), A. israelii (4 of 17; 23.5%), and A. naeslundi (4 of 17; 17.6%). In 7 cases, the species was not specified. In 16 cases, the concomitant microbial flora was noted. In all patients a concomitant mixed anaerobic flora was retrieved. Eight patients (8 of 16; 50.0%) had mixed infections with Streptococcus milleri, and seven patients (7 of 16; 43.8%) had mixed infections with Propionibacterium acnes.

-Treatment

Sixteen (84.2%) patients were treated with intravenous antibiotics, incision and drainage of the swelling, and debridement of necrotic tissue, if needed. Three patients (15.8%) were treated as outpatients with oral AB. Intravenous AB treatment most fre-quently (10 of 19; 52.6%) consisted of penicillin G 12 million units/day with metronidazol 500 mg 3/day. One patient didn’t improve on this AB regimen and was treated with ceftriaxon 2000 mg/day and clindamycin 600 mg 3/day. In case of penicillin allergy, clindamycin would be administered. An overview of treatment and treatment duration is provided in  Table 1.

**Table 1:**
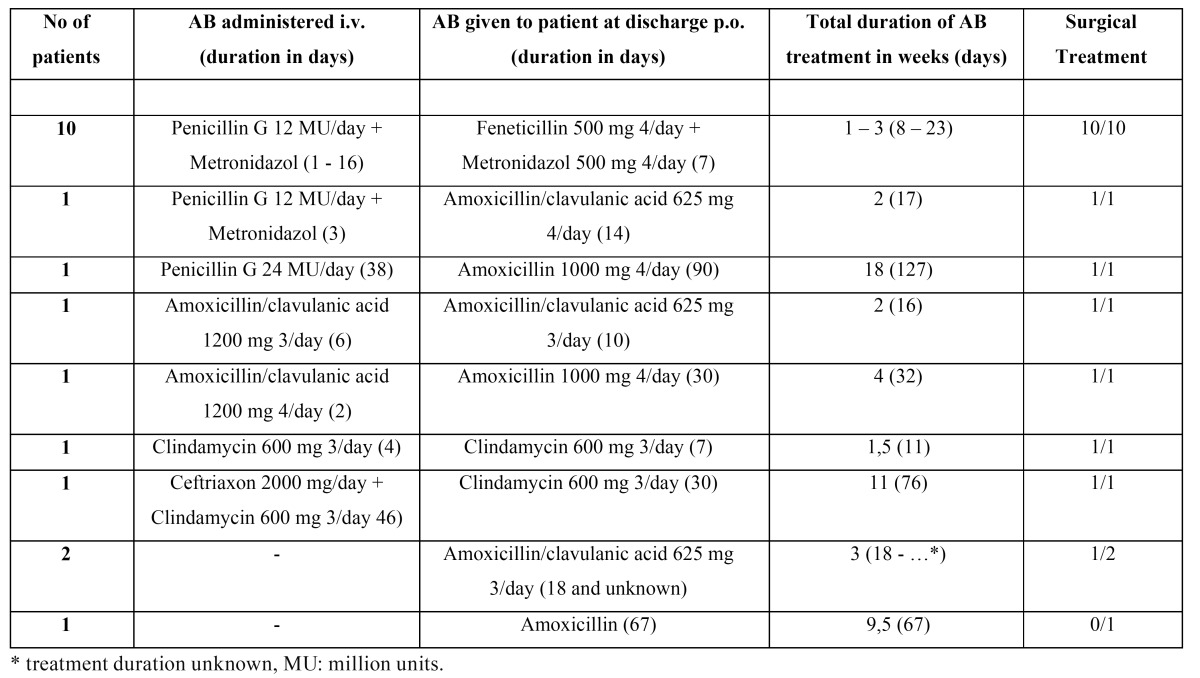
Treatment of cervicofacial actinomycosis in 19 patients.

Patients were discharged from hospital when clinical improvement was seen. Time to clinical improvement ranged from 1 to 46 days (SD 8.4 ± 13.2) when treated with intravenous AB.

-Literature search

**Figure 2:**
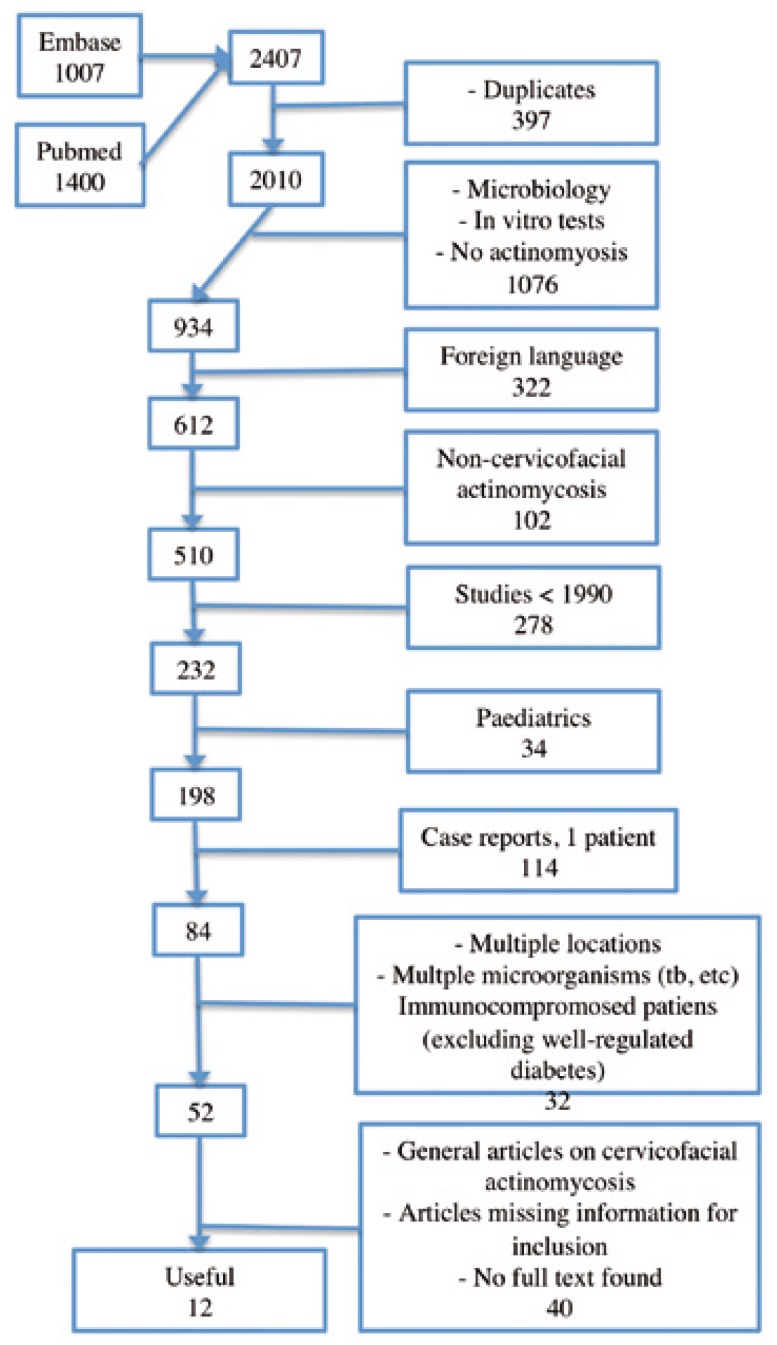
Flow chart of the articles evaluated for inclusion.

Pubmed and Embase were searched, resulting in 1400 and 1007 articles respectively. Of these articles, 397 were duplicates. Relevance was based on information provided in the title and abstract. More than half (1076 of 2010; 53.5%) was not useful. These studies described the microbiology of infections involving primarily other microorganisms than the Actinomyces species, used actinomycosis in the differential diagnosis, or described in vitro tests with Actinomyces species. Of the remaining 934 articles, 322 were of foreign language, 102 involved cases with non-cervicofacial actinomycosis, 278 studies were published before the year 1990, 34 articles described (largely) paediatric patients, 114 reported only one patient, and 32 studies described actinomycosis involving multiple locations, multiple microorganisms (e.g. an Actinomyces and tuberculosis coinfection), and immunocompromised or ill patients (human deficiency virus, poorly regulated diabetes, multiple myeloma, post-transplantation, osteoradionecrosis). Finally, 38 articles were general articles on actinomycosis, not describing patient populations, or articles missing crucial information important for this review. The full text could not be retrieved for two, possibly relevant, articles. Twelve articles remained for inclusion (See fig. 2).

All studies were of retrospective nature. Five studies reported two cases of actinomycosis (12-16). The remaining studies reported cases ranging from 4 to 15 patients (17-23). Custal-Teixidor et al.(17) retrospectively analyzed 15 cases, including a paediatric patient. Treatment of this patient was not mentioned separately, and is included in the current review. Similarly, Bartkowski et al.(18) included 3 patients < 18 years in their analysis of 15 cases. However, the treatment of their patients was reported separately. Thus, the paediatric patients were excluded. Different departments were involved in the treatment of these patients, including the department of otolaryngology head and neck surgery (12,16,19,20,23), dermatology (13), internal medicine (17), Microbioloby (18) plastic and reconstructive surgery (14), radiology (21), and maxillofacial surgery (13,18,22).  Table 2 shows an overview of the treatment modality, treatment duration, and patient outcome retrieved in these 12 publications.

**Table 2:**
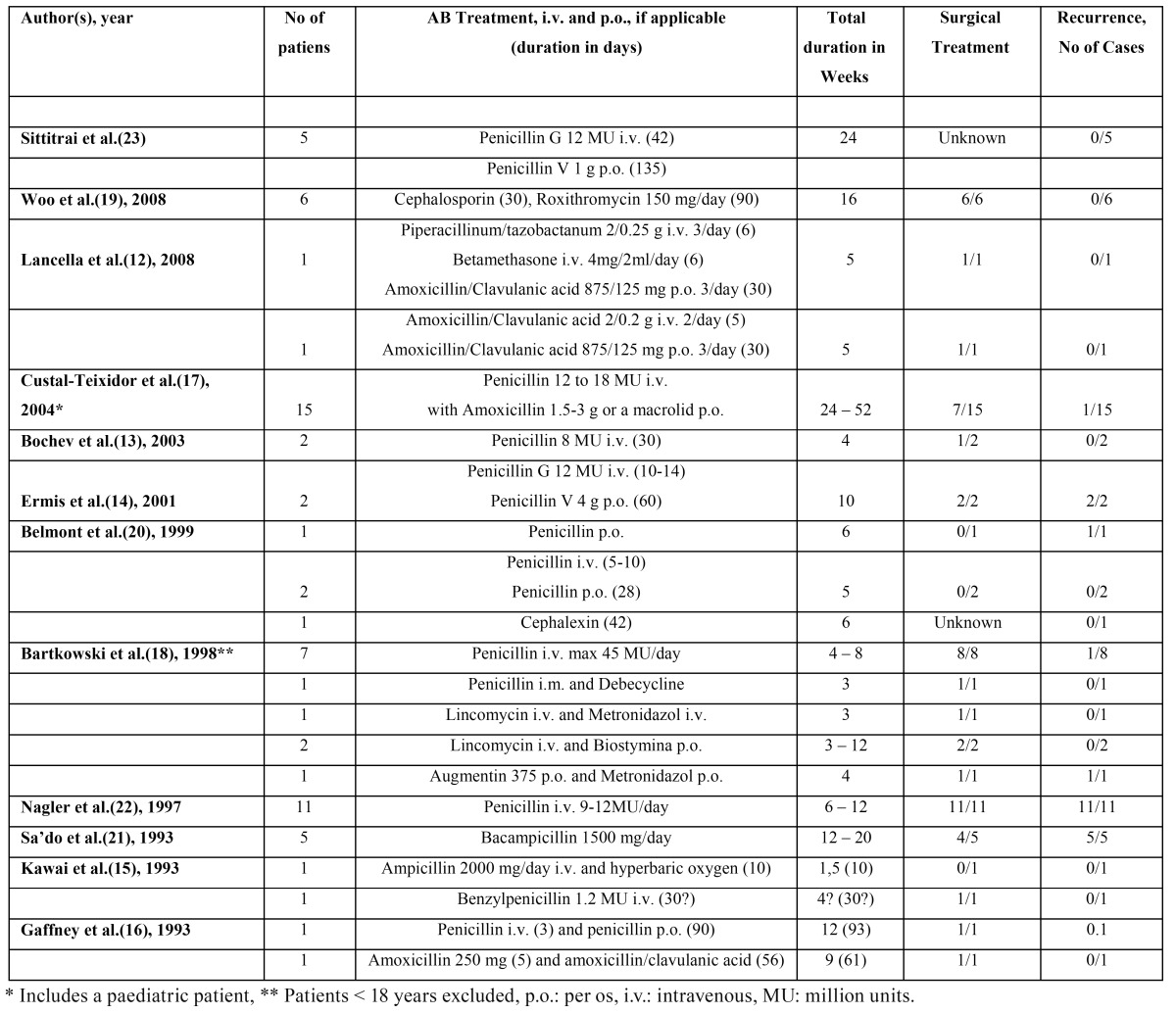
Overview of treatment of cervicofacial actinomycosis in 12 studies.

## Discussion

The current study retrospectively reviewed all patients treated for cervicofacial actinomycosis from January 2000 to December 2010. This provided 19 cases, to our knowledge being the largest case series described since 1990. Actinomycosis can present as a chronic, slowly progressive infiltration, or as a more acute and rapidly progressive swelling (24). In accordance with the literature (7,13,14,16,17,21,22,25) our patients presented with persistent, sometimes recurrent swellings, which were both tender and non-tender, and non-responsive to short-term oral antibiotics. Most studies describe a latency of infection ranging from a few weeks to over one year (12,14-17,20-23,26), whereas most of our patients presented within one week of start of symptoms. This can be explained by the more acute and rapidly progressive nature of the swellings seen in our population. Also, possibly the Dutch health care system encourages a low visiting threshold for patients, and quick referral to specialized clinics, limiting doctor’s delay.

Diagnosis of cervicofacial actinomycosis can be very difficult. In a study performed by Park et al.(7), in 4 out of 7 cases, the swelling was thought to be a malignancy, and in 2 other cases, the swelling was diagnosed as either malignancy or granulomatous disease based on radiologic diagnosis. The suspicion of a malignant mass has also been made by other studies (15,20,21,23). Definitive diagnosis is best made by histopathology after excision, fine needle aspiration or biopsy, revealing the presence of sulfur granules (7,12,13,15,16,19,20). Culturing Actinomyces has proven extremely difficult due to the anaerobic nature of this organism, requiring up to 14 days of strict anaerobic incubation (24). Also, aerobic and anaerobic bacterial overgrowth, or possible suppressive effect of prior antimicrobial therapy might make diagnosis by culture difficult (22). Studies of negative cultures have been reported (16,17,20,22). In these cases diagnosis was made by histopathology. In the current study, all cultures were positive for Actinomyces. The sole explanation for this finding is that this was the inclusion criterion.

Penicillin remains the treatment of choice since the introduction by Nichols and Herrell in 1948 (24). The majority of our patients received antibiotic treatment for a duration of 1 – 4 weeks, being shorter than the 3 – 52 weeks reported in the literature (12-14,16-22). Perhaps this is because our patients were treated with a combination of penicillin and metronidazol, affecting a larger microbial spectrum, and making the environment unfavourable for Actinomyces. Also, intravenous treatment is usually stopped and replaced by oral antibiotics when clinical improvement is seen, preventing prolonged intravenous administration of antibiotics.

Nagler et al. (22) hypothesized that patients with actinomycosis don’t respond well to antibiotic therapy before degranulation and curettage or lesion resection due to compartmentalization of the organisms within the granulation tissue and the sulfur granules. This separates the organism from the blood supply and antibiotics administered. Surgical treatment in our patient population was generally performed shortly after presentation. According to Nagler et al.(22), facilitating the effects of the antibiotic treatment, and, in turn, explaining the short treatment duration needed in our population.

## Conclusions

Actinomyces spp. are normal inhabitants of the oral microbial flora. Thus, a positive culture with Actinomyces spp. does not always imply the diagnosis of actinomycosis. On the other hand, a negative culture does not exclude actinomycosis. Final diagnosis should be made based on clinical findings in combination with bacteriological and/or histopathological findings. When actinomycosis is suspected, our review has shown that a surgical approach in combination with intravenous penicillin and metronidazol until clinical improvement is seen, followed by oral antibiotics for 2 – 4 weeks is generally efficient. Nevertheless, one should always be aware of the indolence of this microorganism and not stop treatment prematurely.
